# CCM and RTAT Malaria Interventions

**DOI:** 10.4269/ajtmh.25-0056

**Published:** 2025-04-22

**Authors:** Richard W. Steketee

**Affiliations:** Independent Consultant, Bethesda, Maryland

In this edition of the *American Journal of Tropical Medicine and Hygiene*, investigators working in Nchelenge District, Luapula Province for many years^1^ describe what they term a “natural crossover design” for evaluating the pre-, during-, and post-program deployment of a combined intervention package of malaria community case management (CCM) and reactive testing and treatment (RTAT) in a high malaria transmission setting in northern Zambia.^2^ For CCM, community health workers (CHWs) were supported across Zambia^3^ and trained to diagnose, triage, treat, and refer patients presenting with fever and suspected malaria. In Nchelenge District, the CHWs were instructed to conduct RTAT, whereby they visited the homes of confirmed malaria cases and neighboring homes within a 140-meter radius to test all residents with an RDT and treat those found to be test-positive. Of note, RTAT may be considered consistent with related WHO guidance, including reactive case detection and treatment (RACDT) or targeted testing and treatment (TTaT).^4^

The authors assembled locally available data to examine the impacts of deploying CCM+RTAT on the reach of services in communities and health facilities and their associated health outcomes including: the numbers of community malaria cases confirmed and treated; disease severity; and survival in hospitalized children. This opportunistic examination included four years of data collection spanning the period before, during, and after the deployment of the CCM+RTAT intervention package.

In fact, the opportunity for this analysis arose because the CCM+RTAT combined intervention “failed”. While the intent of the intervention was understandable and the child health and systems outcomes during the deployment were encouraging, the combined intervention in this high-transmission setting required supplies and a human workload that could not be afforded or sustained. It is rare that a district-wide program can be started and abruptly ended in such a defined and relatively short interval – thus the opportunity to learn.

The district work was evidence of a laudable commitment from the national malaria program, the service delivery systems in Zambia, and their many partners to scale up community-based diagnostic testing and treatment to improve human health. As the authors describe, the Nchelenge District (population ∼242,000 people) is served by one hospital and 15 hospital-linked health facilities. To augment malaria services, the Zambia Ministry of Health trained and deployed 385 CHWs; (approximately 1 per 625 population) in the use of malaria rapid diagnostic tests (RDTs) and malaria treatment with artemether-lumefantrine. Relevant data were available for 14 months prior to the start of CCM+RTAT (starting July 2017), for the 16 months of deployment (starting Sept 2018), and for 18 months after the ending of the CCM+RTAT deployment (starting January 2020). Available data included findings from monthly community surveys, health facility case registers, CHW records, hospital admissions registers, and laboratory data.

There were certainly positive outcomes during the CCM+RTAT deployment including: increased access to care both within localities and through geographic expansion; increased reported healthcare contacts; increased care and treatment provided by CHWs, with a shift of approximately one-third of the malaria case burden from health facilities to CHWs; reduced severity of disease among hospitalized children (e.g., less severe anemia and generally higher hemoglobin levels); and reduced in-hospital mortality (4-fold and 3-fold lower mortality compared to the pre- and post-intervention intervals, respectively). While it is possible that RTAT may have contributed to some of these outcomes, the authors document that other studies of CCM-alone showed similar levels of increased access to care, improved balance of health service systems, and improved morbidity and mortality outcomes.^5^^,^^6^

Why the failure? The authors document that the failure was due to supply requirements for the combined CCM and RTAT components of the intervention package. The study area faced very high and saturated malaria transmission in all age groups, with monthly parasite prevalence averaging 46% to 51% across the evaluation interval, and very little within- or between-year variation despite substantial seasonal changes in temperature, rainfall, and humidity. This high-transmission intensity meant that any community testing and treatment (e.g., during monthly household surveys or during RTAT) could expect approximately one-half of all malaria tests to be positive and require treatment. The monthly parasitemia surveys of ∼1,870 households and ∼7,730 inhabitants might be expected to require over 7,700 RDTs and 3,850 treatments each month. The RTAT efforts reacting to local cases over the 16-month intervention generated 128,592 additional RDT-positive cases requiring treatment; these positives likely came from ∼257,000 RDTs (exceeding the 242,000 district population). Based on study data, during this intervention interval, with each month of deployment, the 385 CHWs identified an average of ∼2,500 cases, and health facilities counted another ∼5,000 cases for a total of ∼7,500 passively-detected cases. The RTAT cases averaged an additional ∼8,000 cases per month. Despite this marked increase in case finding and treatment, community parasite prevalence did not change. Despite substantial CHW training and their September 2018 deployment with supplies, by January of 2020, essentially all malaria testing and treatment by CHWs ended – both for passive and reactive testing in the community. The finality of that ending meant that health facility case loads increased and child hospital admissions reverted to near pre-intervention levels – the benefits were erased, and the system appears to have been scarred by the undoing of the good intent of the program.

What have we learned? National malaria programs in sub-Saharan malaria-endemic countries remain enthusiastic about adding new interventions to reduce the burden of malaria disease and transmission.^7^ The concept of expanding services and access via CHWs remains widely supported in child health programs.^8^^,^^9^ The Global Malaria Technical Strategy 2016–2030 makes a clear call for expanding community outreach to increase access to malaria case management.^10^ At the same time, WHO Global Malaria Programme guidance has cautioned that adding or expanding reactive or targeted testing and treatment beyond the management of the confirmed case will not change malaria transmission in high transmission settings and that RTAT-like interventions should be undertaken only in settings on the verge of or after accomplishing elimination.^4^ On the other hand, there has been much emphasis on the adverse consequences of asymptomatic malaria infections, and it is easy to see how programs want to bypass the caution and introduce a RTAT strategy in higher malaria transmission settings – the impacts of malaria are sufficiently grim that programs are looking for any available options.

While the Zambian decisions to link CHW delivery of combined CCM + RTAT were made with all good intent, this investigation demonstrated that there can be serious consequences to expansion of malaria control services. The cost of adding RTAT to CCM was high, and the consequences of the failure to sustain CCM because of the overwhelming cost of RTAT could devastate programs.

What comes next? The national and local programs and supporting partners in Zambia need to determine how to re-establish expanded malaria community case management to gain its many benefits. Additionally, the program needs additional tools against the parasite and the mosquito vector, and for the human, to further reduce malaria transmission intensity in settings like Nchelenge District, so that elimination locally and more widely in sub-Saharan Africa can be achieved. However, this paper supports the conclusion that RTAT or RTAT-like interventions are not among the needed tools in high-transmission settings.
